# Abnormal Placental Development and Early Embryonic Lethality in EpCAM-Null Mice

**DOI:** 10.1371/journal.pone.0008543

**Published:** 2009-12-31

**Authors:** Keisuke Nagao, Jianjian Zhu, Mallorie B. Heneghan, Jeffrey C. Hanson, Maria I. Morasso, Lino Tessarollo, Susan Mackem, Mark C. Udey

**Affiliations:** 1 Dermatology Branch, Center for Cancer Research, National Cancer Institute, National Institutes of Health, Bethesda and Frederick, Maryland, United States of America; 2 Laboratory of Pathology, Center for Cancer Research, National Cancer Institute, National Institutes of Health, Bethesda and Frederick, Maryland, United States of America; 3 Developmental Skin Biology Unit, National Institute of Arthritis and Musculoskeletal and Skin Diseases, National Institutes of Health, Bethesda, Maryland, United States of America; 4 Mouse Cancer Genetics Program, Center for Cancer Research, National Cancer Institute, National Institutes of Health, Bethesda and Frederick, Maryland, United States of America; 5 Cancer and Developmental Biology Lab, Center for Cancer Research, National Cancer Institute, National Institutes of Health, Bethesda and Frederick, Maryland, United States of America; The University of Hong Kong, China

## Abstract

**Background:**

EpCAM (CD326) is encoded by the *tacstd1* gene and expressed by a variety of normal and malignant epithelial cells and some leukocytes. Results of previous *in vitro* experiments suggested that EpCAM is an intercellular adhesion molecule. EpCAM has been extensively studied as a potential tumor marker and immunotherapy target, and more recent studies suggest that EpCAM expression may be characteristic of cancer stem cells.

**Methodology/Principal Findings:**

To gain insights into EpCAM function *in vivo*, we generated EpCAM −/− mice utilizing an embryonic stem cell line with a *tacstd1* allele that had been disrupted. Gene trapping resulted in a protein comprised of the N-terminus of EpCAM encoded by 2 exons of the *tacstd1* gene fused in frame to βgeo. EpCAM +/− mice were viable and fertile and exhibited no obvious abnormalities. Examination of EpCAM +/− embryos revealed that βgeo was expressed in several epithelial structures including developing ears (otocysts), eyes, branchial arches, gut, apical ectodermal ridges, lungs, pancreas, hair follicles and others. All EpCAM −/− mice died *in utero* by E12.5, and were small, developmentally delayed, and displayed prominent placental abnormalities. In developing placentas, EpCAM was expressed throughout the labyrinthine layer and by spongiotrophoblasts as well. Placentas of EpCAM −/− embryos were compact, with thin labyrinthine layers lacking prominent vascularity. Parietal trophoblast giant cells were also dramatically reduced in EpCAM −/− placentas.

**Conclusion:**

EpCAM was required for differentiation or survival of parietal trophoblast giant cells, normal development of the placental labyrinth and establishment of a competent maternal-fetal circulation. The findings in EpCAM-reporter mice suggest involvement of this molecule in development of vital organs including the gut, kidneys, pancreas, lungs, eyes, and limbs.

## Introduction

Epithelial cell adhesion molecule, EpCAM (CD326), (also termed EGP-2, Egp314, Ep-CAM, GA733-2, gp40, Ly74, panepithelial glycoprotein 314, TROP1, and tumor associated calcium transducer-1 (TACSTD1)), is a putative adhesion molecule. EpCAM was initially described as a cell surface protein that was selectively expressed by epithelial [1]–[3] and some myeloid cancers [4], but it is also expressed in a variety of normal epithelia including skin, thymus, and gut, in adult mice and humans [5]–[7]. Fibroblasts (L cells) that were transiently transfected with cDNA encoding EpCAM formed aggregates in suspension [8], suggesting that EpCAM functioned as a homophilic adhesion molecule. A subsequent study in which L cells were transfected with cDNAs encoding E-cadherin alone or E-cadherin and EpCAM suggested that EpCAM attenuated E-cadherin-mediated adhesion [8], [9]. Localization of EpCAM on polarized epithelial cells *in situ* indicated that the protein accumulated predominantly at intercellular basolateral interfaces that were in close apposition, but that it was excluded from desmosomes and perhaps tight junctions as well [10], [11].

Enrichment of EpCAM in tetraspanin enriched microdomains (TEMs) that contain β1-integrins, CD44v, claudins and tetraspanins [11]–[13] is also consistent with the concept that EpCAM is involved in intercellular adhesion, but TEM-associated proteins have also been implicated in cell signaling and more complex processes including cell migration and metastasis [14]. Indeed, Maetzel et al recently described proteolytic fragments of EpCAM that participated in nuclear signaling in tumor cells [15]. It has also been reported that EpCAM was expressed by stem cells in colon cancers [16] and hepatocellular carcinomas [17], [18]. Studies conducted with human hepatocellular carcinoma cell lines suggested that EpCAM expression distinguished cells with stem-like properties from cells that were more differentiated [18]. In addition, selective knockdown of EpCAM in hepatocellular carcinoma stem cells attenuated their ability to form tumors in immunocompromised mice and, even more strikingly, to metastasize [16], [18]. The extent to which these latter findings might relate to modulation of intercellular adhesion is uncertain.

We became interested in EpCAM after determining that, among dendritic cells, EpCAM was selectively expressed by epidermal Langerhans cells (LC) [5], [19], a distinctive resident skin leukocyte subpopulation with some epithelial characteristics. Because EpCAM function is incompletely characterized, and the functional activities of dendritic cells are best studied *in vivo*, we sought to explore the function of this molecule in EpCAM-deficient mice. We identified an embryonic stem (ES) cell line with an EpCAM (*tacstd1*) mutation created by gene trapping, and used it to generate EpCAM-knockout mice which we then characterized. EpCAM −/− mice exhibited early embryonic lethality with markedly abnormal placental development. Although many mechanistic aspects of EpCAM function remain to be elucidated, these results indicate that this protein is critical for normal placental development and plays one or more non-redundant roles in normal epithelial physiology.

## Materials and Methods

### Generation of EpCAM-Deficient Mice

A mouse ES cell line containing an inserted gene trapping construct in *tacstd1* (RST556, strain 129P2), the gene encoding EpCAM, was developed by BayGenomics (San Francisco, CA), a consortium of research groups supported by the National Heart, Lung and Blood Institute [20]. The gene-trap vector used (pGT0, 1, 2tm-pfs) contained a splice-acceptor sequence, en-2, upstream of a transmembrane domain that is fused in frame with the reporter gene/selectable marker βgeo (itself a fusion protein with β-galactosidase and neomycin phosphotransferase II activities) [20]. Using 5′ rapid amplification of cDNA ends [21], we verified that the insertional mutation in RST556 was located in the third intron of *tacstd1*. This mutation resulted in the production of an in frame fusion transcript consisting of exons 1 (untranslated region), 2, and 3 from *tacstd1*, a CD2-derived transmembrane domain, and βgeo. ES cells were microinjected into C57BL/6 recipient blastocysts to generate chimeras that transmitted the mutated allele to progeny in the Center for Cancer Research Knockout Mouse Core Facility [22]. EpCAM +/− mice were then inter-crossed to obtain homozygous deficient animals. For determination of embryonic ages, noon on the day of the post-coital plug was taken to be E0.5. Wild type and heterozygous littermates were used as controls as indicated. All mice were bred and housed in a pathogen-free environment and used in experiments in accordance with institutional guidelines at the Center for Cancer Research, National Cancer Institute, National Institutes of Health. All experimental procedures conducted in this study were approved by the Animal Care and Use Committee, Center for Cancer Research, National Cancer Institute, National Institutes of Health.

### Genotyping

The following primers were used for genotyping: 5′-GCTCCAAACGTGAGTAAATCAATC-3′ (EpCAM wild-type forward; WT 1), 5′-TGGAAAGGCGATGACAGTAACG-3′ (EpCAM wild-type reverse; WT 2) for the wild-type allele, and 5′- CACTCCAACCTCCGCAAACT-3′ (EpCAM mutant reverse; KO 1) in conjunction with the wild-type forward primer to detect the mutant allele. Because the primer pair that amplified βgeo gave identical results but yielded more robust signals, the following primers were subsequently used to detect mutant alleles: 5′-TTCACTGGCCGTCGTTTTACAACGTCGTGA- 3′ (βgeo forward; KO 2), 5′-ATGTGAGCGAGTAACAACCCGTCGGATTCT-3′ (βgeo reverse; KO 3).

### β-galactosidase Staining

To ascertain EpCAM expression patterns, whole embryos were stained with a β-galactosidase staining kit (Invitrogen) in accordance with the manufacturer's instructions. In brief, embryos were fixed in 2% glutaldehyde/2% paraformaldehyde for 15–60 min depending on the age of embryo, stained overnight with X-gal solution, and post-fixed with 4% PFA.

### Laser Capture Microdissection

Embryonic tissue was acquired from formalin-fixed, paraffin-embedded tissue sections via laser capture microdissection (LCM) using the Veritas microdissection system (MDS Analytical Technologies) in the Center for Cancer Research Laser Capture Microdissection core facility. Genomic DNA was purified with QIAamp DNA micro kits (Qiagen) and genotypes were determined by PCR.

### Immunofluorescence Microscopy

The following antibodies were used for immunofluorescence microscopy: anti-EpCAM (10 µg/ml; clone G8.8, Developmental Hybridoma Bank, University of Iowa), anti-E-cadherin (10 µg/ml; ECCD-2, Invitrogen), anti-P-cadherin (10 µg/ml; PCD-1, Invitrogen), and anti-PL-1 (10 µg/ml; Santa Cruz Biotech). The polyclonal anti-Dlx3 antibody was described previously [23]. Antigen retrieval was performed as described [24]. Briefly, formalin-fixed, paraffin-embedded tissue sections were deparaffinized and subjected to 5×2 min cycles of microwave treatment in citrate buffer (pH 6) and allowed to cool to room temperature. Antigen-retrieved or frozen sections were blocked with 3% milk (Bio-Rad) and 5% goat or donkey serum in addition to mouse IgG (10 µg/ml) for 1 hr at room temperature. Primary antibodies were incubated overnight at 4°C, sections were washed, and bound antibodies were detected with AlexaFluor-labeled anti-goat, anti-rabbit, or anti-rat secondary antibodies (5 µg/ml; Invitrogen). Stained sections were mounted in Prolong Gold with DAPI (Invitrogen) and analyzed. Immunofluorescence images were collected and visualized with an AxioImager A1 imunofluorescence microscope (Zeiss) and Axiovision software version 4.6 (Zeiss). Intensity levels of digital images in experimental and control specimens were adjusted proportionally and in the linear range either in Axiovison or Photoshop CS2 (Adobe).

#### *In situ* hybridization

A 950 bp EpCAM cDNA fragment was inserted into BamHI-EcoRI sites of pcDNA3 (Invitrogen). Antisense Digoxigenin-labeled RNA probes were synthesized with BamHI-linearized vector and Sp6 RNA polymerase. Whole mount *in situ* hybridization was carried out using an established protocol [25] with a short proteinase K treatment (2 µg/ml proteinase K for 5 min).

## Results

### Generation of Mice with a Mutated *tacstd1* Allele

Generation of knockout mice by gene trapping involves random insertion of a construct that includes an splice acceptor that efficiently “traps” one or more upstream exons by incorporating them into a fusion protein encoded by a cDNA immediately downstream of the splice acceptor site. In the vector used in the present study, the cDNA downstream of the splice acceptor site encodes a portion of CD2 (inclusive of the transmembrane domain) in frame with a fusion protein that retains β-galactosidase and neomycin phosphotransferase activities (βgeo). Because expression of the fusion protein that results from gene trapping is expected to occur only in cells in which the targeted locus is being actively transcribed, the pattern and intensity of expression of βgeo can be studied as a surrogate marker for expression of the protein encoded by the targeted gene.

**Figure 1:**
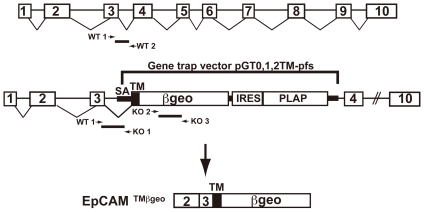
Disruption of the *tacstd1*gene that encodes murine EpCAM. The schematic indicates the site of insertion of the gene trapping vector as well as the structure of the mRNA that encodes the EpCAM/βgeo fusion protein. Locations of primers used for genotyping are also depicted.

After searching the Baygenomics database (see International gene trap consortium, http://www.genetrap.org/), we identified four ES cell lines that were predicted to include the gene trapping vector inserted in the gene encoding EpCAM (*tacstd1*). For this study, we utilized ES cell line RST556 that contained the trapping construct inserted into the third intron of *tacstd1* (Fig. 1). A chimeric male founder mouse was generated and crossed to normal C57BL/6 mice to generate heterozygous mice containing one copy of the mutated EpCAM allele. Mice were genotyped using PCR as described in the Materials and Methods. EpCAM +/− mice were viable, healthy, grossly indistinguishable from their wildtype littermate counterparts, and were fertile.

### Expression of EpCAM in Multiple Organs during Development

**Figure 2:**
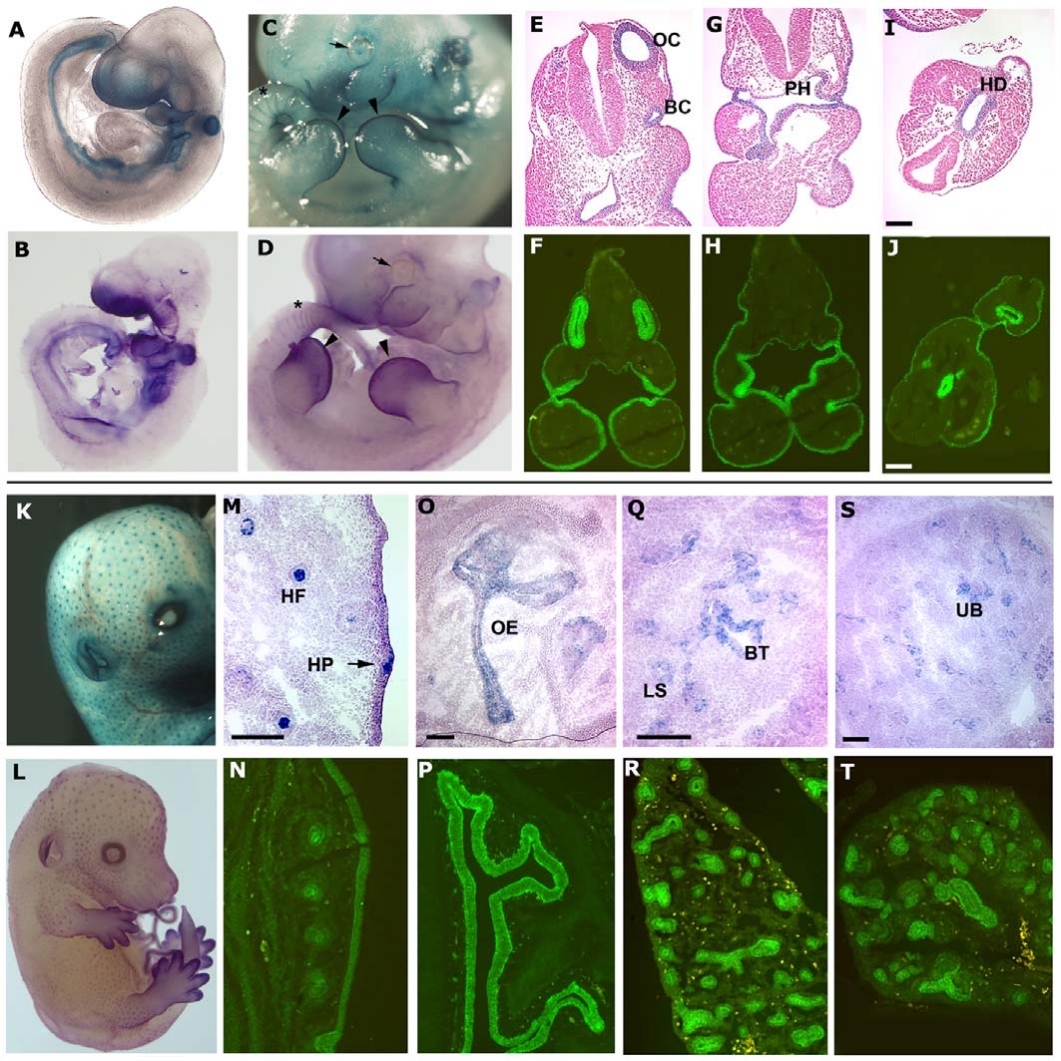
Expression of EpCAM during embryonic development. (A–D) Whole mount LacZ staining (A&C, in blue) and in situ hybridization (B&D, in purple) of E9.5 (A&B) and E11.5 (C&D) embryos. EpCAM expression in the apical ectodermal ridges (arrowheads), around the eyes (arrows) and inter-somitic regions (asterisks) is highlighted in C and D. Corresponding transverse sections with LacZ and light H&E staining (E, G, I), and immunofluorescence staining using rat anti-mouse EpCAM mAb (F, H, J, in green). OC = otocyst, BC = 1^st^ branchial cleft, Ph = pharynx, HD = hindgut diverticulum. (K–T) EpCAM expression in epithelia in a variety of organs at E14.5. K, M, O, Q, and S show LacZ and N, P, R, T show immunofluorescence staining. Primordial hair follicles are prominently stained in the E14.5 embryo as determined by LacZ staining (K&M), in situ hybridization (L), and immunofluorescence (N). M to T shows EpCAM expression in skin (M&N), nasal plexus (O&P), lungs (Q&R), and kidneys (S&T). Bars = 100 µm. HF = hair follicle, HP = hair placode, OE = olfactory epithelium, BT = bronchiolar tubules, LS = lung saccules, UB = ureteric bud branches.

Evaluation of EpCAM expression using LacZ staining at E8.5 revealed a pattern that was predominantly endodermal (data not shown), consistent with recent reports that indicated that EpCAM can be used as a marker to enrich endodermal cells from early embryos [26]. At E9.5, EpCAM expression was most prominent in the facial primordia, gut, branchial arches, and otocyst (Fig. 2A, B). Corresponding transverse sections revealed concordant expression of EpCAM as demonstrated by LacZ or immunofluorescence staining using anti-EpCAM antibody (Fig. 2E–I, F–J). At E11.5 and earlier, the periocular region, the apical ectodermal ridge precursors (ventral limb ectoderm) and mature ridge, and inter-somitic regions in the posterior trunk/tail stained positive (Fig. 2C, D). Particularly prominent expression was evident in developing hair follicles in whole mounts prepared at E14.5 (Fig. 2K–N). In analogy to what has previously been reported in rats [27], EpCAM expression was readily detected in some epithelial components of a variety of organs including hair follicles, the nasal plexus, lungs, kidneys, and pancreas examined at E14.5 (Fig. 2K–T, Table 1).

**Table 1:** EpCAM expression patterns in various organs during embryonic development.

Embryonic stage	EpCAM-expressing tissues
E9.5	Ear, brachial arch, pharynx, gut
E11.5	Limb ventral ectoderm, apical ectodermal ridge, ear, whole GI tract, intersomitic junction, eye, pharynx
E14.5	Ear, hair follicle, limb ectoderm, eye, nasal/turbinate, lung, kidney, pancreas, GI tract

### Embryonic Lethality in EpCAM-Deficient Mice

**Figure 3:**
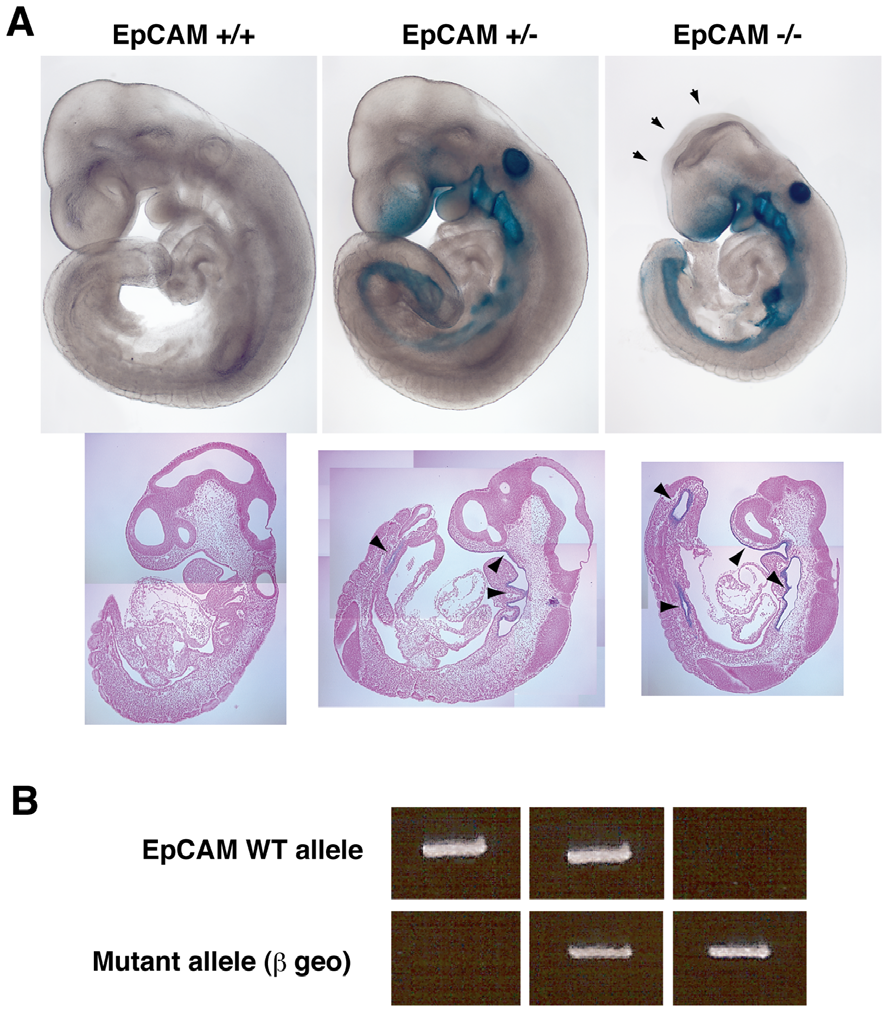
Developmental delay in EpCAM-deficient embryos. (A) Embryonic development in wild type, haplosufficient and EpCAM-deficient littermate embryos (E9.5). Arrows indicate open neural tube. Sagittal sections show EpCAM (βgeo) expression in gut endoderm in EpCAM +/− and EpCAM −/− embryos (arrowheads). The embryos depicted were littermates and photos of whole mounts were taken at identical magnifications. (B) Genotypes of embryos depicted in (A).

EpCAM +/− mice were intercrossed to generate homozygous deficient animals. No EpCAM −/− pups were identified in >24 litters born to >10 breeding pairs (see Table 2). Examination of embryos of various gestational ages revealed that EpCAM −/− embryos appeared to develop normally up to E8.5, after which development arrested, and became nonviable during E10.5-E11.5 (Table 3). EpCAM−/− embryos with gestational ages ≥E12.5 were not identified. At E9.5, EpCAM −/− embryos were significantly smaller than EpCAM-sufficient siblings and sometimes exhibited delayed, incomplete neural tube closure (Fig. 3). Localization of βgeo in whole mounts and sagittal sections of E9.5 EpCAM-deficient embryos revealed intense staining of otocysts, branchial arches and gut.

**Table 2:** Distribution of genotypes in viable pups resulting from matings of EpCAM +/− mice.

		EpCAM genotype		
Litter Number	Number of viable pups	+/+	+/−	−/−
1	7	1	6	0
2	2	0	2	0
3	6	2	4	0
4	5	3	2	0
5	10	4	6	0
6	5	3	2	0
7	10	6	4	0
8	14	4	10	0
9	5	4	1	0
10	2	1	1	0
11	2	0	2	0
12	12	5	7	0
13	5	1	4	0
14	7	4	3	0
15	4	2	2	0
16	3	1	2	0
17	5	3	2	0
18	4	2	2	0
19	5	1	4	0
20	4	0	4	0
21	3	1	2	0
22	7	3	4	0
23	8	4	4	0
24	5	3	2	0
Total Number (%)	140 (100%)	58 (41%)	82 (59%)	0 (0%)

**Table 3:** Time course of lethality in EpCAM −/− mice.

		EpCAM genotype			
Age	N =	+/+	+/−	−/−	[dead]
E9.25-9.75	36 (4)*	7	13	16	[0]
E10.5-10.75	42 (6)*	10	22	10†	[5]
E11.5	28 (5)*	9	10	9†	[5]
E12.5	14 (3)*	5	9	0	

† most remaining live embryos developmentally arrested at E10.5-10.5.

* number of litters examined in parentheses.

### EpCAM Expression in Developing Placenta

**Figure 4:**
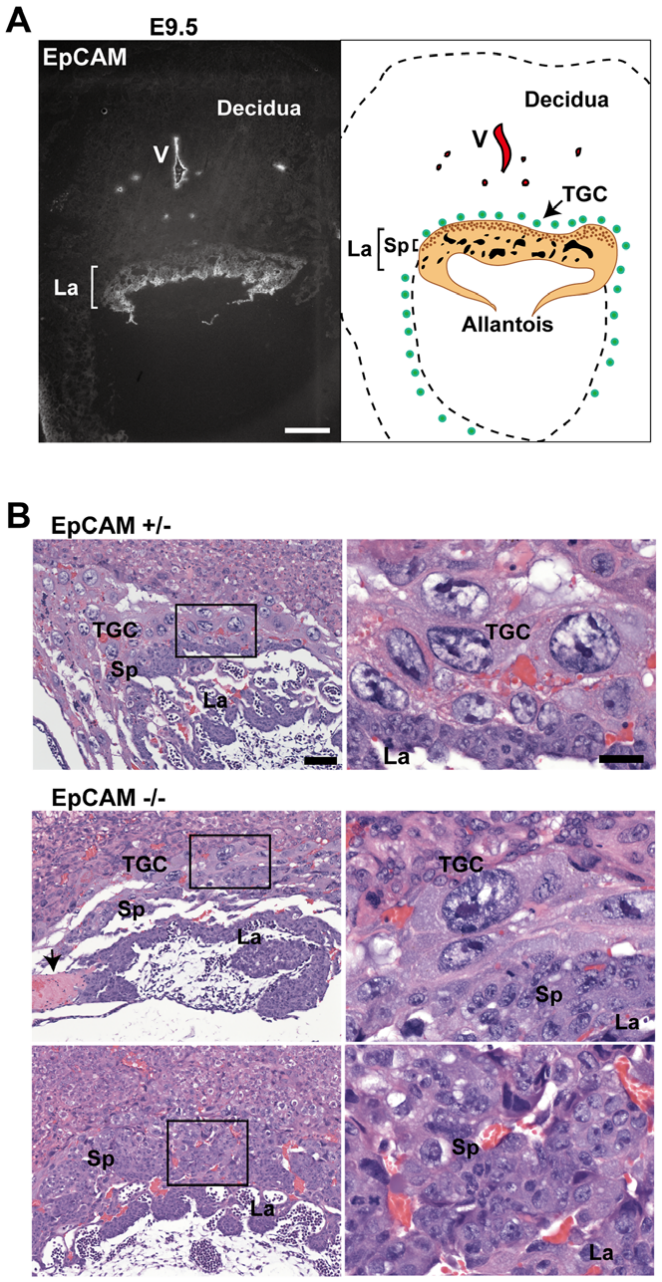
Defective placental development in the absence of EpCAM. (A) EpCAM expression in wild type C57BL/6 placentas at E 9.5 as shown by immunofluorescence staining (left). Bar = 200 µm. Right panel shows a schematic of E9.5 placenta. La = labyrinth, Sp = spongiotrophoblasts, TGC = parietal trophoblast giant cells, V = vasculature. (B) Morphology in H&E stained sections of EpCAM +/− and −/− placentas. Bars = 50 and 20 µm. Panels on the right represent high power view of insets in the left panels. Arrow in B highlights intravascular microthrombus.

**Figure 5:**
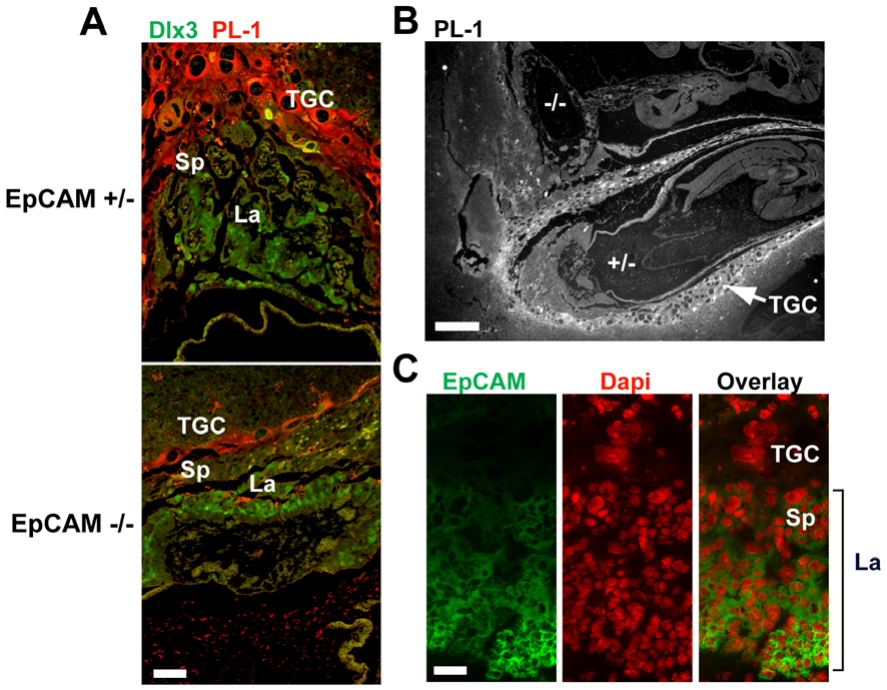
Diminished parietal trophoblast giant cell population in EpCAM −/− placentas. (A) Sections of E9.5 placenta double-stained with anti-Dlx3 antibody and anti-PL-1 antibody after antigen retrieval. (B) Anti-PL-1 staining of a section that contained two embryos (EpCAM −/−, upper embryo; EpCAM +/−, lower embryo) in one E9.5 uterine decidual swelling. (C) EpCAM and DAPI staining of a frozen section of an E9.5 wild type placenta. Bars = 50 (A), 200 (B), and 20 µm (C).

Although EpCAM was widely expressed in developing embryos, the early embryonic lethality that we detected suggested the possibility of an extra-embryonic cause. To address this possibility, we examined placentas for evidence of EpCAM expression. Localization of EpCAM in frozen tissue from E8.5 and E9.5 placentas using immunofluorescence microscopy revealed expression in the allantois, the labyrinthine layer, and in spongiotrophoblasts as well (Fig. 4A & 5C). The highest levels of expression were detected in the labyrinth. Interestingly, vascular structures in the maternal decidua also stained strongly for EpCAM.

To preserve spatial relationships and obtain optimal placental morphology, we embedded embryos *in utero* and prepared sections from formalin-fixed, paraffin-embedded tissues. Placentas were subsequently genotyped using embryo-derived genomic DNA that was obtained from adjacent sections using laser-capture microdissection. E8.5 EpCAM −/− extra-embryonic tissues showed no obvious abnormality (data not shown). As shown in Fig. 4B, whereas well-developed labyrinthine layers with obvious villi were present in E9.5 EpCAM +/− placentas, placentas from EpCAM −/− mice were small, the labyrinthine layer was thin and villous formation was limited. EpCAM −/− placentas were also notable for markedly decreased frequencies of parietal trophoblast giant cells. In addition, micro-thrombi were observed in surrounding capillaries adjacent to the chorionic plate in some EpCAM −/− placentas, suggesting impaired fluid flow or hypercoagulability.

### EpCAM −/− Placentas Contain Decreased Numbers of Parietal Trophoblast Giant Cells

To better characterize cellular constituents of EpCAM −/− placentas, tissue sections of E9.5 placentas were stained for placental lactogen-1 (PL-1) and Dlx3, markers that identify parietal trophoblast giant cells and the labyrinth, respectively [28]. PL-1 positive parietal trophoblast giant cells were normally represented in EpCAM +/− placentas, but they were dramatically reduced in number in EpCAM-deficient tissue (Fig. 5A). The differences were particularly striking in an example of fraternal twin embryos, one EpCAM −/− and the other EpCAM +/−, in a single uterine decidual swelling (Fig. 5B). Consistent with the morphology observed in hematoxylin- and eosin-stained sections (Fig. 4B), the Dlx3-expressing labyrinth layer thickness was markedly reduced in EpCAM −/− embryos (Fig. 5A). Dlx3 was clearly expressed, however, and the levels of expression in EpCAM −/− tissue may exceed that present in normal placentas. To directly address the issue of EpCAM expression by parietal trophoblast giant cells, we stained frozen sections with anti-EpCAM antibodies and DAPI. Interestingly, EpCAM was not detected on parietal trophoblast giant cells (Fig. 5C).

### Distribution of EpCAM and Cadherins in Developing Placenta

**Figure 6:**
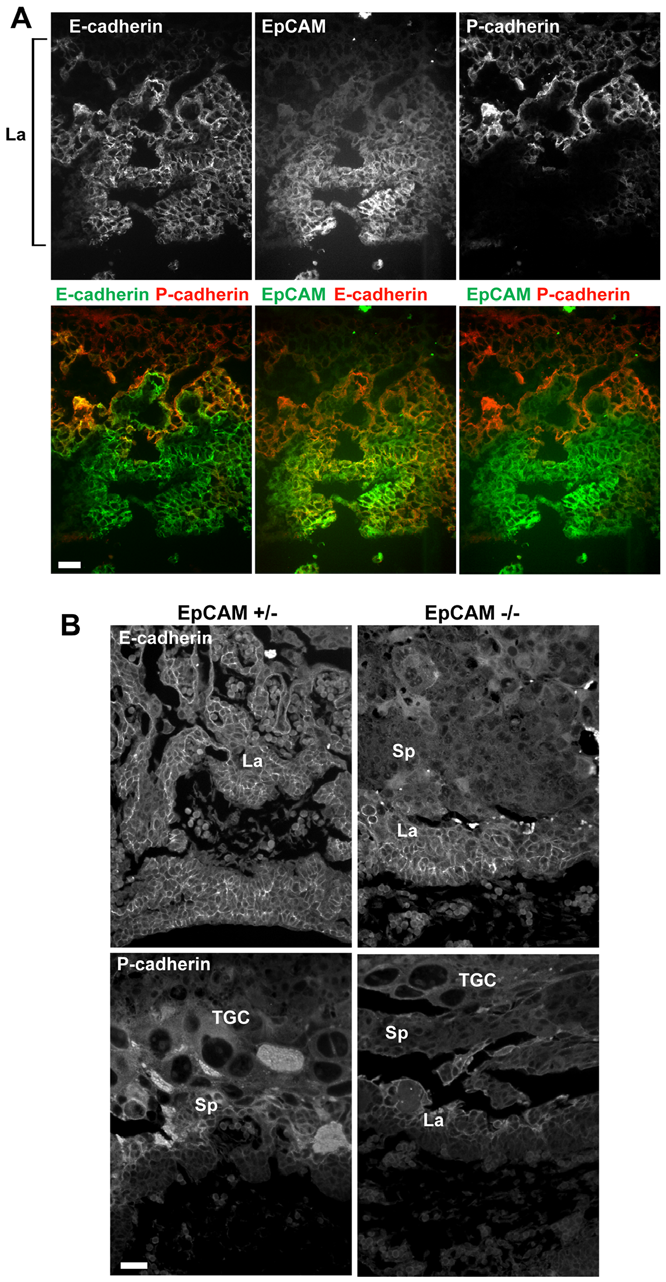
Cadherin expression in EpCAM-sufficient and deficient placentas. (A) Frozen sections from E9.5 wild type placentas stained for EpCAM, E-, and P-cadherins (La = labyrinth). (B) Sections from formalin-fixed, paraffin-embedded tissues stained for E-cadherin and P-cadherin after antigen retrieval. Bars = 20 µm.

Because previous studies suggested that EpCAM might modulate cadherin function [10], [29], we characterized E9.5 placentas for expression of EpCAM, E-cadherin and P-cadherin. EpCAM was co-expressed with E-cadherin throughout the labyrinth, with the highest levels of EpCAM expression occurring on cells in closest proximity to the embryonic chorionic plate (Fig. 6A). In striking contrast, expression of EpCAM was inversely correlated with P-cadherin expression, which was selectively expressed by spongiotrophoblasts. E-cadherin and P-cadherin were reciprocally expressed in the labyrinth, but appeared to be co-expressed in the spongiotrophoblast layer.

To determine if placental cadherin expression might be perturbed in the absence of EpCAM, we stained formalin-fixed, paraffin-embedded sections from EpCAM +/− and EpCAM −/− placentas for E- and P-cadherin after antigen retrieval. Although the labyrinth was clearly abnormal in EpCAM −/− placentas, E-cadherin was expressed throughout (Fig. 6B). P-cadherin distribution was not obviously different in EpCAM-deficient as compared with EpCAM-sufficient placentas.

### Relationships between EpCAM and Cadherin Expression in Other Tissues

The co-expression of EpCAM and E-cadherin and the reciprocal expression that we observed with P-cadherin in placenta was striking. To determine if these relationships were maintained in EpCAM-expressing epithelia in other developing organs, we surveyed skin, gut and lung in E14.5 mice using immunofluorescence microscopy. We observed that E-cadherin and EpCAM were concordantly expressed in developing gut, and P-cadherin and EpCAM were reciprocally expressed (Fig. S1A). In developing lung, most (but not all) E-cadherin-expressing developing bronchi also expressed P-cadherin (Fig. S1B). Those that did not express P-cadherin stained for EpCAM, and there was no overlap between EpCAM and P-cadherin expression. In developing hair follicles, EpCAM and E-cadherin were concordantly expressed by superficial epithelial cells, while P-cadherin staining was most intense on basal cells (Fig. S1C).

## Discussion

We generated EpCAM-deficient mice to gain additional insights into EpCAM function *in vivo*. We utilized a pre-existing ES stem cell clone [20], [30] with a targeted allele producing a fusion protein that contained a short N-terminal portion of EpCAM in addition to βgeo. Consistent with results previously reported in rat embryos [27], we determined that EpCAM was expressed in a variety of epithelial structures, including apical ectodermal ridges, signaling centers that are essential for limb bud outgrowth. In all epithelial tissues examined, EpCAM and E-cadherin were expressed by the same cells, whereas EpCAM was reciprocally expressed with P-cadherin.

No viable EpCAM −/− pups were obtained. Examination of embryos obtained after timed heterozygote matings revealed that −/− embryos implanted, and that control and EpCAM-deficient embryos could not be distinguished on or before E8.5. By E9.5, however, EpCAM knockout embryos were smaller than EpCAM sufficient embryos, neural tube closure was often delayed, and all EpCAM knockout embryos became nonviable before E12.5. Our observation of prominent EpCAM expression in the early placenta prompted us to look for extraembryonic abnormalities. Studies of implantation sites *in situ* revealed that placentas associated with EpCAM −/− embryos were small and thin, the latter characteristic reflecting more compact labyrinthine layers that did not exhibit prominent villous formation or vascularity. Numbers of parietal trophoblast giant cells were also strikingly diminished in EpCAM-deficient placentas. Localization of EpCAM in E8.5 and E9.5 placentas revealed that the protein was highly expressed by trophoblasts in the labyrinthine layer and also by spongiotrophoblasts. EpCAM was not detected on parietal trophoblast giant cells, suggesting the possibility of a nonautonomous effect on giant cell number.

In light of previous studies implicating EpCAM as a modulator of E-cadherin-mediated adhesion [9], [29], it is interesting to compare the phenotypes of EpCAM- and cadherin-deficient mice. E-cadherin-deficient embryos do not form trophectoderm or blastocysts and do not implant [31], [32]. Although both E-cadherin and EpCAM are widely expressed in early embryos and in developing epithelia, post-implantation placental abnormalities were the most striking changes identified in the setting of EpCAM deficiency. P-cadherin is abdundant in placenta and is frequently expressed in developing and adult epithelia, but P-cadherin-deficient mice were viable, fertile, and they did not exhibit obvious epithelial abnormalities [33]. It has been suggested that the ability of E-cadherin to compensate for loss of P-cadherin in many tissues may explain the unexpectedly mild phenotype of P-cadherin-deficient mice. Clearly, EpCAM has one or more important functions that cannot be compensated by other proteins, including E-cadherin.

The embryonic lethality of EpCAM deficiency may reflect an as yet uncharacterized global effect on epithelial cell physiology, or a requirement for EpCAM in the development or survival of a critical placental cell type. The diminished frequency of parietal trophoblast giant cells is among the most striking histologic abnormalities in EpCAM-deficient placentas. Parietal trophoblast giant cells are the first cells to differentiate from the trophoectoderm, but they may also derive from trophoblasts and spongiotrophoblasts via endoreplication [34]. Parietal trophoblast giant cells invade into the uterine wall, establishing a receptive environment for development of a functional maternal-fetal interface via elaboration of paracrine factors including VEGF, metalloproteinases, hormones and cytokines [35], [36]. Alternatively, the embryonic lethality seen in our study may be secondary to parietal trophoblast giant cell deficiency, since null mutations in *Hand1* (a basic helix-loop-helix transcription factor that enhances parietal trophoblast giant cell development) and *I-mfa* (an inhibitor of *Mash2*, a basic helix-loop-helix protein that inhibits parietal trophoblast giant cell development) both lead to parietal trophoblast giant cell deficiency, arrested placental development and embryonic lethality [37], [38]. Because EpCAM-deficient placentas were smaller and the labyrinthine layers were thinner and less elaborate than in controls, impaired placental development in this setting may reflect a more global effect on epithelial cell physiology. EpCAM is expressed by embryonic stem cells [39] and cancer stem cells [16], [17], [40], and it is possible that EpCAM influences trophoblast stem cell behavior.

In future studies, it will be of interest to explore other potential roles of EpCAM *in vivo*. These studies will require generation of mice that carry EpCAM alleles that can be targeted in selected lineages and/or at selected times. The importance of elucidating EpCAM function *in vivo* is highlighted by recent studies that demonstrate that at least some cases of congenital tufting enteropathy, a rare, typically autosomal recessive syndrome that is characterized by intractable infantile diarrhea, result from mutations in the gene encoding EpCAM [41]. Intrauterine demise or abnormal fertility has not been reported in families of patients with congenital tufting enteropathy, but it may be significant that null mutations in the gene encoding EpCAM, or mutations that resulted in truncated EpCAM protein, have not yet been identified in patients. The mutations identified to date may be hypomorphs that result in identifiable abnormalities in some EpCAM-expressing epithelia (eg intestinal epithelia and perhaps ocular conjunctiva), but not others. The availability of conditional knockout mice and transgenic mice that express candidate hypomorphic EpCAM alleles will allow EpCAM function to be additionally explored in embryonic development, reproduction, and adult physiology. Recent experiments in zebrafish implicate EpCAM in epithelial morphogenesis during epiboly and skin development [42], suggesting that future studies of conditional EpCAM knockout mice will be informative.

## Supporting Information

Figure S1 Co-expression of EpCAM and E-cadherin and reciprocal expression with P-cadherin in epithelia in developing non-placental tissues. EpCAM, E-cadherin and P-cadherin were stained in frozen sections from E14.5 embryos in the (A) gut, (B) lungs (BT = bronchiolar tubules, LS = lung saccules), (C) and hair follicles. EpCAM and E-cadherin expression is present in gut epithelium, and P-cadherin is expressed in muscular/serosal layers in (A). Bars = 100 µm (A&B), and 20 µm (C).
(7.94 MB TIF)

## References

[1] Edwards DP, Grzyb KT, Dressler LG, Mansel RE, Zava DT (1986). Monoclonal antibody identification and characterization of a Mr 43,000 membrane glycoprotein associated with human breast cancer.. Cancer Res.

[2] Herlyn M, Steplewski Z, Herlyn D, Koprowski H (1979). Colorectal carcinoma-specific antigen: detection by means of monoclonal antibodies.. Proc Natl Acad Sci U S A.

[3] Ross AH, Herlyn D, Iliopoulos D, Koprowski H (1986). Isolation and characterization of a carcinoma-associated antigen.. Biochem Biophys Res Commun.

[4] Bergsagel PL, Victor-Kobrin C, Timblin CR, Trepel J, Kuehl WM (1992). A murine cDNA encodes a pan-epithelial glycoprotein that is also expressed on plasma cells.. J Immunol.

[5] Borkowski TA, Nelson AJ, Farr AG, Udey MC (1996). Expression of gp40, the murine homologue of human epithelial cell adhesion molecule (Ep-CAM), by murine dendritic cells.. Eur J Immunol.

[6] Nelson AJ, Dunn RJ, Peach R, Aruffo A, Farr AG (1996). The murine homolog of human Ep-CAM, a homotypic adhesion molecule, is expressed by thymocytes and thymic epithelial cells.. Eur J Immunol.

[7] Trzpis M, McLaughlin PMJ, de Leij LMFH, Harmsen MC (2007). Epithelial cell adhesion molecule: more than a carcinoma marker and adhesion molecule.. Am J Pathol.

[8] Litvinov SV, Velders MP, Bakker HA, Fleuren GJ, Warnaar SO (1994). Ep-CAM: a human epithelial antigen is a homophilic cell-cell adhesion molecule.. J Cell Biol.

[9] Winter M (2003). Expression of Ep-CAM shifts the state of cadherin-mediated adhesions from strong to weak.. Exp Cell Res.

[10] Balzar M, Briaire-de Bruijn IH, Rees-Bakker HA, Prins FA, Helfrich W (2001). Epidermal growth factor-like repeats mediate lateral and reciprocal interactions of Ep-CAM molecules in homophilic adhesions.. Mol Cell Biol.

[11] Ladwein M, Pape U, Schmidt D, Schnolzer M, Fiedler S (2005). The cell-cell adhesion molecule EpCAM interacts directly with the tight junction protein claudin-7.. Exp Cell Res.

[12] Kuhn S, Koch M, Nübel T, Ladwein M, Antolovic D (2007). A complex of EpCAM, claudin-7, CD44 variant isoforms, and tetraspanins promotes colorectal cancer progression.. Mol Cancer Res.

[13] Schmidt D, Klingbeil P, Schnolzer M, Zoller M (2004). CD44 variant isoforms associate with tetraspanins and EpCAM.. Exp Cell Res.

[14] Hemler ME (2005). Tetraspanin functions and associated microdomains.. Nat Rev Mol Cell Biol.

[15] Maetzel D, Denzel S, Mack B, Canis M, Went P (2009). Nuclear signalling by tumour-associated antigen EpCAM.. Nat Cell Biol.

[16] Dalerba P, Dylla SJ, Park I-K, Liu R, Wang X (2007). Phenotypic characterization of human colorectal cancer stem cells.. Proc Natl Acad Sci U S A.

[17] Yamashita T, Budhu A, Forgues M, Wang XW (2007). Activation of hepatic stem cell marker EpCAM by Wnt-beta-catenin signaling in hepatocellular carcinoma.. Cancer Res.

[18] Yamashita T, Forgues M, Wang W, Kim JW, Ye Q (2008). EpCAM and alpha-fetoprotein expression defines novel prognostic subtypes of hepatocellular carcinoma.. Cancer Res.

[19] Nagao K, Ginhoux F, Leitner WW, Motegi SI, Bennett C (2009). Murine epidermal Langerhans cells and langerin-expressing dermal dendritic cells are unrelated and exhibit distinct functions.. Proc Natl Acad Sci U S A.

[20] Stryke D, Kawamoto M, Huang CC, Johns SJ, King LA (2003). BayGenomics: a resource of insertional mutations in mouse embryonic stem cells.. Nucleic Acids Res.

[21] Townley DJ, Avery BJ, Rosen B, Skarnes WC (1997). Rapid sequence analysis of gene trap integrations to generate a resource of insertional mutations in mice.. Genome Res.

[22] Bonin A, Reid SW, Tessarollo L (2001). Isolation, microinjection, and transfer of mouse blastocysts.. Methods Mol Biol.

[23] Hwang J, Mehrani T, Millar SE, Morasso MI (2008). Dlx3 is a crucial regulator of hair follicle differentiation and cycling.. Development.

[24] Peralta Soler A, Knudsen KA, Tecson-Miguel A, McBrearty FX, Han AC (1997). Expression of E-cadherin and N-cadherin in surface epithelial-stromal tumors of the ovary distinguishes mucinous from serous and endometrioid tumors.. Hum Pathol.

[25] Pizard A, Haramis A, Carrasco AE, Franco P, Lopez S (2004). Whole-mount in situ hybridization and detection of RNAs in vertebrate embryos and isolated organs.. Curr Protoc Mol Biol.

[26] Sherwood RI, Jitianu C, Cleaver O, Shaywitz DA, Lamenzo JO (2007). Prospective isolation and global gene expression analysis of definitive and visceral endoderm.. Dev Biol.

[27] Stephan JP, Roberts PE, Bald L, Lee J, Gu Q (1999). Selective cloning of cell surface proteins involved in organ development: epithelial glycoprotein is involved in normal epithelial differentiation.. Endocrinology.

[28] Morasso MI, Grinberg A, Robinson G, Sargent TD, Mahon KA (1999). Placental failure in mice lacking the homeobox gene Dlx3.. Proc Natl Acad Sci U S A.

[29] Litvinov SV, Balzar M, Winter MJ, Bakker HA, Briaire-de Bruijn IH (1997). Epithelial cell adhesion molecule (Ep-CAM) modulates cell-cell interactions mediated by classic cadherins.. J Cell Biol.

[30] Skarnes W, Moss J, Hurtley S, R.S.P B (1995). Capturing genes encoding membrane and secreted proteins important for mouse development.. Proc Natl Acad Sci U S A.

[31] Larue L, Ohsugi M, Hirchenhain J, Kemler R (1994). E-cadherin null mutant embryos fail to form a trophectoderm epithelium.. Proc Natl Acad Sci U S A.

[32] Riethmacher D, Brinkmann V, Birchmeier C (1995). A targeted mutation in the mouse E-cadherin gene results in defective preimplantation development.. Proc Natl Acad Sci U S A.

[33] Radice Gl, Mc F, Robinson Sd (1997). Precocious mammary gland development in P-cadherin-deficient mice.. J Cell Biol.

[34] Simmons DG, Fortier AL, Cross JC (2007). Diverse subtypes and developmental origins of trophoblast giant cells in the mouse placenta.. Dev Biol.

[35] Bany BM, Cross JC (2006). Post-implantation mouse conceptuses produce paracrine signals that regulate the uterine endometrium undergoing decidualization.. Dev Biol.

[36] Simmons DG, Cross JC (2005). Determinants of trophoblast lineage and cell subtype specification in the mouse placenta.. Dev Biol.

[37] Kraut N, Snider L, Chen CM, Tapscott SJ, Groudine M (1998). Requirement of the mouse I-mfa gene for placental development and skeletal patterning.. Embo J.

[38] Riley P, Anson-Cartwright L, Cross JC (1998). The Hand1 bHLH transcription factor is essential for placentation and cardiac morphogenesis.. Nat Genet.

[39] Anderson R, Schaible K, Heasman J, Wylie C (1999). Expression of the homophilic adhesion molecule, Ep-CAM, in the mammalian germ line.. J Reprod Fertil.

[40] Dan YY, Riehle KJ, Lazaro C, Teoh N, Haque J (2006). Isolation of multipotent progenitor cells from human fetal liver capable of differentiating into liver and mesenchymal lineages.. Proc Natl Acad Sci U S A.

[41] Sivagnanam M, Mueller JL, Lee H, Chen Z, Nelson SF (2008). Identification of EpCAM as the gene for congenital tufting enteropathy.. Gastroenterology.

[42] Slanchev K, Carney TJ, Stemmler MP, Koschorz B, Amsterdam A (2009). The epithelial cell adhesion molecule EpCAM is required for epithelial morphogenesis and integrity during zebrafish epiboly and skin development.. PLoS Genet.

