# Supplementary figures and images for: Abnormal Placental Development and Early Embryonic Lethality in EpCAM-Null Mice
Source: PLoS One. 2009 Dec 31;4(12):e8543. doi: 10.1371/journal.pone.0008543 (PMC2796178; doi:10.1371/journal.pone.0008543)

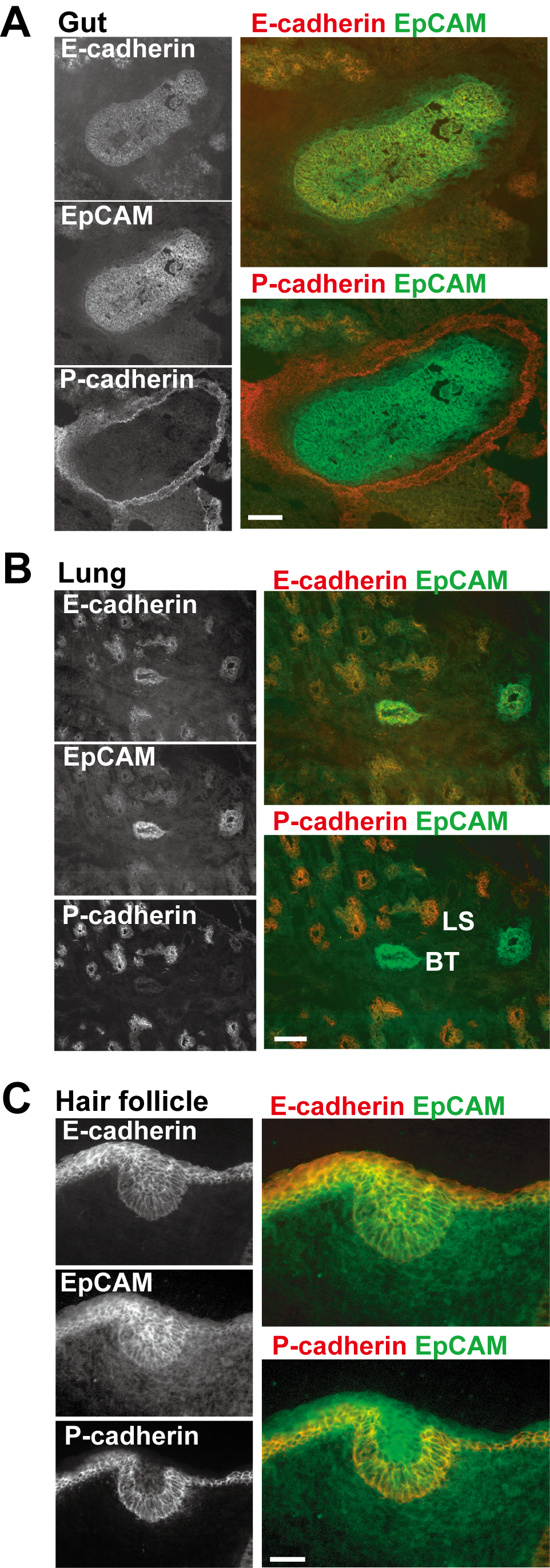

Supplement: Figure S1 — Co-expression of EpCAM and E-cadherin and reciprocal expression with P-cadherin in epithelia in developing non-placental tissues. EpCAM, E-cadherin and P-cadherin were stained in frozen sections from E14.5 embryos in the (A) gut, (B) lungs (BT = bronchiolar tubules, LS = lung saccules), (C) and hair follicles. EpCAM and E-cadherin expression is present in gut epithelium, and P-cadherin is expressed in muscular/serosal layers in (A). Bars = 100 µm (A&B), and 20 µm (C). (7.94 MB TIF) [file pone.0008543.s001.tif]
